# Novel lipid mediator 7*S*,14*R*-docosahexaenoic acid: biogenesis and harnessing mesenchymal stem cells to ameliorate diabetic mellitus and retinal pericyte loss

**DOI:** 10.3389/fcell.2024.1380059

**Published:** 2024-03-12

**Authors:** Yan Lu, Haibin Tian, Hongying Peng, Quansheng Wang, Bruce A. Bunnell, Nicolas G. Bazan, Song Hong

**Affiliations:** ^1^ Neuroscience Center of Excellence, School of Medicine, L.S.U. Health, New Orleans, LA, United States; ^2^ Tongji University, Shanghai, China; ^3^ Biostatistics, Department of Environmental Health, University of Cincinnati College of Medicine, Cincinnati, OH, United States; ^4^ Tongji Medical College, Huazhong University of Science and Technology, Wuhan, Hubei, China; ^5^ Tulane University School of Medicine, Center for Stem Cell Research and Regenerative Medicine, New Orleans, LA, United States; ^6^ Department of Ophthalmology, School of Medicine, L.S.U. Health, New Orleans, LA, United States

**Keywords:** diabetic mellitus and complications, mesenchymal stem cell, 7S,14R-diHDHA, blood glucose level, β-cell and α-cell, insulin, pancreas and islets, retinopathy

## Abstract

**Introduction:** Stem cells can be used to treat diabetic mellitus and complications. ω3-docosahexaenoic acid (DHA) derived lipid mediators are inflammation-resolving and protective. This study found novel DHA-derived 7*S*,14*R*-dihydroxy-4*Z*,8*E*,10*Z*,12*E*,16*Z*,19*Z*-docosahexaenoic acid (7*S*,14*R*-diHDHA), a maresin-1 stereoisomer biosynthesized by leukocytes and related enzymes. Moreover, 7*S*,14*R*-diHDHA can enhance mesenchymal stem cell (MSC) functions in the amelioration of diabetic mellitus and retinal pericyte loss in diabetic *db/db* mice.

**Methods:** MSCs treated with 7*S*,14*R*-diHDHA were delivered into *db/db* mice *i.v.* every 5 days for 35 days.

**Results:** Blood glucose levels in diabetic mice were lowered by 7*S*,14*R*-diHDHA-treated MSCs compared to control and untreated MSC groups, accompanied by improved glucose tolerance and higher blood insulin levels. 7*S*,14*R*-diHDHA-treated MSCs increased insulin^+^ β-cell ratio and decreased glucogan^+^ α-cell ratio in islets, as well as reduced macrophages in pancreas. 7*S*,14*R*-diHDHA induced MSC functions in promoting MIN6 β-cell viability and insulin secretion. 7*S*,14*R*-diHDHA induced MSC paracrine functions by increasing the generation of hepatocyte growth factor and vascular endothelial growth factor. Furthermore, 7*S*,14*R*-diHDHA enhanced MSC functions to ameliorate diabetes-caused pericyte loss in diabetic retinopathy by increasing their density in retina in *db/db* mice.

**Discussion:** Our findings provide a novel strategy for improving therapy for diabetes and diabetic retinopathy using 7*S*,14*R*-diHDHA-primed MSCs.

## 1 Introduction

Type 2 diabetes, accounting for > 90% of diabetes worldwide, is a major public health problem and places a severe economic burden on healthcare systems. It is characterized by insulin resistance in peripheral tissues. As long as pancreatic β-cells are able to compensate for insulin resistance by enhancing insulin secretion and increasing β-cell mass, euglycemia can be maintained. However, when they are unable to meet the body’s demand for insulin because of genetic defects and/or exogenous insults, diabetes mellitus ensues (Gunton et al., 2005; Florez, 2008; Cerf, 2013; Dludla et al., 2023). Chronic hyperglycemia leads to glucose toxicity and worsening of impaired insulin secretion due to the overworking of pancreatic β-cells, which results in a decreased ability to secrete insulin. Hyperglycemia can also cause oxidative stress, which influences β-cell survival (Tiedge et al., 1997; Eguchi et al., 2021). Reduced β-cell function has been observed in both obese and lean type 2 diabetic humans and in diabetic rodent models (Pick et al., 1998; Butler et al., 2003; Puff et al., 2011; Inaishi and Saisho, 2020; Miranda et al., 2020). Therefore, prevention of the progression of pancreatic β-cell dysfunction in patients with diabetes mellitus should be a key in the long-term management of diabetes mellitus.

Hypoglycemic agents can significantly reduce the development of diabetic complications (1998; Want, 2008; Group et al., 2022); however, many of them cause adverse effects and sometimes result in progressive deterioration in β-cell function (Efanova et al., 1998; Hakobyan et al., 2011; Dalle et al., 2023). Recently, some strategies using antioxidative treatment have been shown to ameliorate diabetic mellitus. An angiotensin II type 1 receptor blocker was shown to decrease oxidative stress markers in β-cells, improve abnormalities of pancreatic islets in diabetes *db/db* mice (Shao et al., 2006; Chen et al., 2018; Lee et al., 2023). Also, increasing levels of the antioxidant enzyme, glutathione peroxidase-1, specifically in β-cells, has been demonstrated to protect them against the adverse effects of chronic hyperglycemia in *db/db* mice (Harmon et al., 2009; Robertson, 2023).

Multiple differentiation capacity, paracrine functions, and immunomodulation action make the marrow mesenchymal stem cells (MSCs) a good candidate for prevention or cure of diabetic mellitus and complications (Vija et al., 2009; Bernardi et al., 2012; Miklosz and Chabowski, 2023; Aglan et al., 2024). Transplantation of bone marrow–derived cells increased levels of serum insulin and reduced blood glucose levels in hyperglycemic mice with streptozotocin-induced pancreatic tissue damage, which improved the metabolic state and survival of recipients (Hess et al., 2003; Zhoujun et al., 2023). Studies using rat MSCs (Boumaza et al., 2009; Yousef et al., 2022) or human bone marrow–derived multipotent stromal cells (Lee et al., 2006; Gabr et al., 2013; Carlsson et al., 2023) have shown similar results of enhanced insulin secretion and repair of pancreatic islets after streptozotocin treatment in rodents. Patients with type 2 diabetes received an intrapancreatic autologous bone marrow stem cell infusion; after 1 year, these patients showed improved metabolic control and reduced insulin requirements (Wang et al., 2011; Mathur et al., 2023). However, diabetic hyperglycemia and concomitant oxidative stress damage DNA, proteins, and lipids in various tissues, resulting in dysfunction of cells and enzyme systems. When MSCs are transplanted into diabetes, the inhospitable environment might damage MSC functions (Kornicka et al., 2018; Yin et al., 2021).

Diabetic retinopathy, a secondary microvascular complication of diabetes mellitus, is the leading cause of blindness in the United States among individuals aged 20 to 64 (Bhavsar, 2006; Gange et al., 2021). A persistent increase in blood glucose levels shunts excess glucose into the aldose reductase pathway in certain tissues, which converts sugars into alcohol (e.g., glucose into sorbitol, galactose to dulcitol). Pericytes of retinal capillaries seem to be affected by this increased level of sorbitol. Thus, the loss of function of pericytes results in weakness and eventual vascular outpouching of capillary walls, resulting in microaneurysms (Bhavsar, 2006; Eshaq et al., 2017).

Docosahexaenoic acid (DHA) is an essential omega-3 fatty acid. Resolvin D series, neuroprotectins, and maresins are potent anti-inflammatory lipid mediators (LMs) naturally generated from DHA during inflammation and/or resolution of inflammation (Serhan et al., 2002; Hong et al., 2003; Marcheselli et al., 2003; Antony et al., 2010; Spite and Serhan, 2010; Cortina et al., 2013; Pham and Bazan, 2021). Neuroprotectin D1 and other DHA derived lipid mediators have been reported to protect retinal epithelial cells and neuronal-glial cells from apoptosis induced by oxidative stress (Hong et al., 2003; Mukherjee et al., 2004; Zhao et al., 2011; Hong et al., 2014b), promote neuronal functions and prevent neural degeneration (Bazan, 2006; Asatryan and Bazan, 2017; Pham et al., 2017; Emre et al., 2021; Emre et al., 2022). We identified a DHA-derived LM, 14*S*,21*R*-diHDHA, which promotes wound healing (Lu et al., 2010; Tian et al., 2011a), inhibits MSC apoptosis, and enhances MSC functions to ameliorate acute kidney injury (Tian et al., 2012). In 2009, Serhan *et al.* reported their discovery of maresin 1 (7*R*,14*S*-dihydroxy-4*Z*,8*E*,10*E*,12*Z*,16*Z*,19*Z*-DHA and its inflammation-resolving functions (Serhan et al., 2009). Inspired by this discovery, we identified and prepared a novel maresin 1 stereoisomer, 7*S*,14*R*-dihydroxy-4*Z*,8*E*,10*Z*,*12E*,16*Z*,19*Z*-DHA (7*S*,14*R*-diHDHA) from leukocytes and related enzyme incubations, as we predicted the therapeutic significance that the biofunction of maresin 1 is conserved in this stereoisomer and that it could promote MSC amelioration of multiple diseases and injuries. Maresins promote the beneficial bioactions of stem cells in the treatment of diseases (Cianci et al., 2016; Albuquerque-Souza et al., 2020; Martinez-Fernandez et al., 2021; AlZahrani et al., 2022; Rakian et al., 2022; Sugimoto et al., 2022; Yao et al., 2022; Walker et al., 2024). Maresin 1 regulates insulin signaling in human adipocytes as well as in adipose tissue and muscle of lean and obese mice (Martinez-Fernandez et al., 2021). It inhibits hyperglycemia-induced ferroptosis (Li et al., 2023). DHA preserves visual function by maintaining correct disk morphology in retinal photoreceptor cells (Shindou et al., 2017).

Accordingly, we hypothesized that MSCs can protect pancreas β-cells from the damage of diabetic oxidative stress and ameliorate retinal pericyte loss, and biogenic 7*S*,14*R*-diHDHA can induce MSC protective functions. This was further tested to be positive by our studies *in vitro* and by our experiments using *db/db* diabetic mouse model *in vivo*. 7*S*,14*R*-diHDHA was formed by the cytochrome P450 enzyme and 5-lipoxygenase (5-LOX) in tandem and by leukocytes. 5-LOX catalyzed the formation of 7*S*-hydroxyl in DHA and 5-LOX is the major lipoxygenase in leukocytes (el Makhour-Hojeij et al., 1994; Cook-Moreau et al., 2007; Radmark and Samuelsson, 2009; Serhan et al., 2009). 7*S*,14*R*-diHDHA induced MSC functions in amelioration of diabetes mellitus in *db/db* mice by lowering blood sugar and increasing glucose tolerance and blood insulin levels, and decreased the number of macrophages in islets, increasing β-cell viability, density and insulin secretion, and reducing pericyte loss in the retina. 7*S*,14*R*-diHDHA can enhance the MSC secretion of trophic growth factors. Overall, pretreatment of MSCs with 7*S*,14*R*-diHDHA, or related lipid mediators or structural mimics, may offer a new clinical strategy for improved treatment of diabetic mellitus and retinopathy as well as other diabetic complications.

## 2 Materials and methods

### 2.1 Reagents

Racemic ±14-hydroxy-4*Z*,7*Z*,10*Z*,12*E*,16*Z*,19*Z*-DHAs (±14-HDHAs or 14*S/R*-HDHAs) and 5-lipoxygenase (5-LOX, human recombinant or potato) were supplied by Cayman (Ann Arbor, MI, United States). NaBH_4_, interleukin (IL)-1β, TNF-α, and *Escherichia coli* lipopolysaccharide (LPS) were supplied by Sigma-Aldrich (St. Louis, MO, United States).

### 2.2 Cells

MSCs were isolated from the bone marrow of *C57BL/6J* mice (8 weeks old, female, Jackson Laboratory), as described in our publications (Tian et al., 2012); more than 95% of isolated MSCs were positive for Sca-1 and CD29 based on flow cytometric analysis (Izadpanah et al., 2006; Tian et al., 2011a; Tian et al., 2012). These MSCs possessed adipogenic and chondrogenic differentiation ability. They were cultured in Dulbecco’s modified Eagle’s medium (DMEM) containing 10% mesenchymal supplement (Stem Cell Technologies, Vancouver, BC, Canada). Cells in passages 7 through 10 were used for the experiments. The Min6 mouse β-cell line (ATCC, Manassas, VA, United States) was cultured in RPMI1640 containing 10% fetal bovine serum (ATCC, Manassas, VA, United States).

### 2.3 Isolation of human neutrophils, monocytes, and lymphocytes

Human samples were managed following protocols approved by the Institutional Review Boards of our institutes. Established procedures were followed (Serhan and Romano, 1995; Hong et al., 2003). Human peripheral blood supplied by the Blood Center (New Orleans, LA, United States) was from a healthy donor who was unknown to us and who had not been on medication for > 2 weeks prior to donation. We centrifuged the blood in sodium citrate (180 g*,* 10 min, 23°C); then centrifuged the supernatants (1,100 g, 15 min, 23°C). The plasma (the top layer) was removed. We washed the cell pellets with 7 mM EDTA. Neutrophils and mononuclear cells were separated from blood by a Ficoll-Hypaque gradient. We plated mononuclear cells onto plastic dishes to further separate the cells. The cells adhered to the dishes were monocytes, and nonadhered cells were lymphocytes. Neutrophils, monocytes, and lymphocytes prepared were of > 96% purity. Cell viability was > 95% according to trypan blue exclusion.

### 2.4 Biogenesis of novel 7*S*, 14*R*-DHA

14*R*-hydroxy-4*Z*,7*Z*,10*Z*,12*E*,16*Z*,19*Z*-DHA (14*R*-HDHA) was prepared from ±14-HDHAs as described in our previous publication (Hong et al., 2014a) and the section below. We then incubated the 14*R*-HDHA (10 µg/incubation) with 5-LOX (3 h, 37°C) to identify the biogenic pathway of the novel maresin-1 stereoisomer, 7*S*, 14*R* dihydroxyl DHA, which is expected to be produced by 5-LOX with 14*R*-hydroxyl preserved. A few grains of NaBH_4_ powder were added at the end of incubation. For cellular generation of 7*S*,14*R*-diHDHA, human leukocytes (6 × 10^6^ neutrophils + 5 × 10^5^ monocytes + 3 × 10^6^ lymphocytes) were incubated (20 min, 37°C) in phosphate-buffered saline (PBS) containing 200 μg 14*R*-HDHA. This ratio of leukocytes resembles the typical leukocyte profile of healthy adults (Lee et al., 2018; Cheng et al., 2022). The cells were then stimulated (37°C, 30 min) with inflammatory factors (10 ng/mL TNFα, 10 ng/mL IL-1β, and 100 ng/mL LPS) as described in our previous publication, to promote the biosynthesis of lipid mediators (Lu et al., 2010; Tian et al., 2011a; Hong et al., 2014a).

### 2.5 Analysis and isolation of 7*S*, 14*R*-diHDHA and of its precursor 14*R*-HDHA

14*R*-HDHA (>98% pure) was isolated from purchased ±14-HDHAs using aqueous reversed-phase chiral liquid chromatography (aR chiral LC) coupled to an ultraviolet photo diode array detector and tandem mass spectrometer (aR chiral LC-UV-MS/MS) as we published previously (Lu et al., 2010; Tian et al., 2011a; Hong et al., 2014a). The incubations were extracted and then analyzed or fractionated for 7*S*,14*R*-diHDHA. Briefly, A Chiralpak-IA chiral column (150 mm long x 2.1 mm ID x 5 μm, Chiral Tech, West Chester, PA, United States) was used for the aR chiral-LC separation. The mobile phase flowed at 0.2 mL/min, eluted as B (methanol:H_2_O:acetic acid = 27:73:0.01) from 0 to 1 min, ramped from B:methanol 40:60 to B:methanol 20:80 by 50 min, ramped to methanol by 55 min, and then flowed as methanol. The instrument used included the system of Survey LC-PDA UV-LTQ linear ion trap MS/MS (Thermo Fisher, Waltham, MA, USA) (Lu et al., 2010; Tian et al., 2011a) and the system of an Agilent 1100 LC system (HPLC-PDA, Agilent Technologies, Santa Clara, CA, United States) and a QTRAP 6500^+^ quadruple-linear trap MS/MS (Sciex.com, Framingham, MA, United States), with electrospray ionization for both systems (Baravkar et al., 2024). The LC-UV effluents were split at a 1:20 ratio, where 1 portion went into a mass spectrometer that monitored the chromatography of 7*S*,14*R*-diHDHA and 20 portions were fraction-collected manually for the aR chiral LC peak of 7*S*,14*R*-diHDHA. The collected effluents containing 7*S*,14*R*-diHDHA were pooled together and evaporated for solvent removal under vacuum or nitrogen gas blow.

### 2.6 Protocols for the treatment of cells *in vitro*

MSCs (2 × 10^5^) were treated with 7*S*,14*R*-diHDHA (250 nM) for 12 h. The level of VEGF or HGF in supernatant was analyzed using ELISA kits (RayBiotech, Peachtree Corners, GA, United States, and R&D Systems, United States, respectively). Min6 β-cells were cultured to 60%–80% confluence (1.8 × 10^5^) in lower chamber of a 24-transwell plate (8 µm pore) pretreated with polylycine overnight. MSCs (3 × 10^4^) were cultured in the upper chamber of a 24-transwell pretreated with or without 250 nM 7*S*,14*R*-diHDHA for 4 h Min6 β-cells and MSCs were cocultured in RPMI1640 for 8 h, and then treated with 100 or 200 µM H_2_O_2_ for 10 h. Cell viability was analyzed using the MTT method. Moreover, Min6 β-cells were treated for 4 h with conditioned medium (CM) of MSCs or 7*S*,14*R*-diHDHA-treated MSCs.

### 2.7 Mice

We used 16-week-old female *C57BL/6J* mice and diabetic *db/db* mice (BKS.Cg-m+/+leprdb/J, Strain #:000642) (Jackson Lab, Bar Harbor, ME). Animal protocols were approved by the Institutional Animal Care and Use Committee and Institutional Review Board of Louisiana State University Health Sciences Center, New Orleans, and followed the ARRIVE guidelines (Percie du Sert et al., 2020). The studies were conducted in a blinded fashion.

### 2.8 Treatment procedures *in vivo*

MSCs were cultured with or without 250 nM 7*S*,14*R*-diHDHA for 4 h and then collected trypsinization (0.25% trypsin/0.53 mM ethylenediaminetetraacetic acid; Invitrogen, Carlsbad, CA, United States), similar to the procedures that we used previously (Tian et al., 2012). MSCs (1 × 10^6^) with or without treatment of 7*S*,14*R*-diHDHA were infused into 16-week-old *db/db* mice via tail vein every 5 days for 35 days. During the intervention period, body weight was measured every week. Nonfasting blood glucose was measured using a OneTouch Ultra glucose meter (LifeScan, Milpitas, CA, United States) every week. The intraperitoneal glucose tolerance test (IPGTT) was conducted as follows. After 5 weeks of treatment, mice were injected intraperitoneally with glucose (2 g/kg body weight) after 8 h of fasting. Blood samples were taken at 0, 60, and 120 min from the tail vein, and blood glucose was measured. Sera were collected at 120 min, and insulin concentrations were measured using ELISA using mouse insulin as a standard.

### 2.9 Histological study

Mice were perfused with PBS at the end of the treatment. Pancreas and eyes were collected and fixed in 4% paraformaldehyde for 2 h, and cryosections (5 µm) were prepared. Sections were blocked with normal goat serum (10% in PBS) for 30 min at room temperature, and then incubated with the following primary antibodies: rabbit antimouse insulin, rabbit antimouse glucagon, rat antimouse F4/80 (Santa Cruz Biotechnology, Dallas, Texas, United States), and rabbit antimouse α-SMA (Millipore, Burlington, MA, United States). Primary antibodies were followed by incubation with the secondary antibodies: Cy3-labeled goat antirat immunoglobulin G (IgG), Cy5-, or fluorescein isothiocyanate (FITC)-labeled goat antirabbit IgG (Invitrogen, Carlsbad, California, United States). Normal rat IgG and rabbit IgG (Santa Cruz Biotechnology, Dallas, Texas, United States) were used as isotype controls. Nuclei were counterstained with Hochest 33342 (Invitrogen, Carlsbad, California, United States). To assess the α cell ratio, the image analysis software NIH ImageJ was used to calculate the pancreatic islet cell number, the insulin^+^ and glucagon^+^ cells in the pancreatic islets to determine the percent β-cell count and α-cell count (relative to the pancreatic islets). The macrophage infiltration in the pancreas was assessed using the number of F4/80^+^ cells per hpf. To assess retinal pericyte loss, retinal sections were stained with an antibody against α-SMA, which specifically marks pericytes in the capillaries. The number of α-SMA^+^ cells in the inner nuclear layer of the retinas was counted and normalized against the area of the inner nuclear layer.

### 2.10 Statistical analysis

All data were analyzed using one-way analysis of variance followed by Fisher’s least significant difference *post hoc* test and expressed as mean ± standard error of the mean. *p* < 0.05 was considered statistically significant.

## 3 Results

### 3.1 Biogenesis of novel 7*S*,14*R*-dihydroxy-4*Z*,8*E*,10*Z*,12*E*,16*Z*,19*Z*-DHA

**FIGURE 1:**
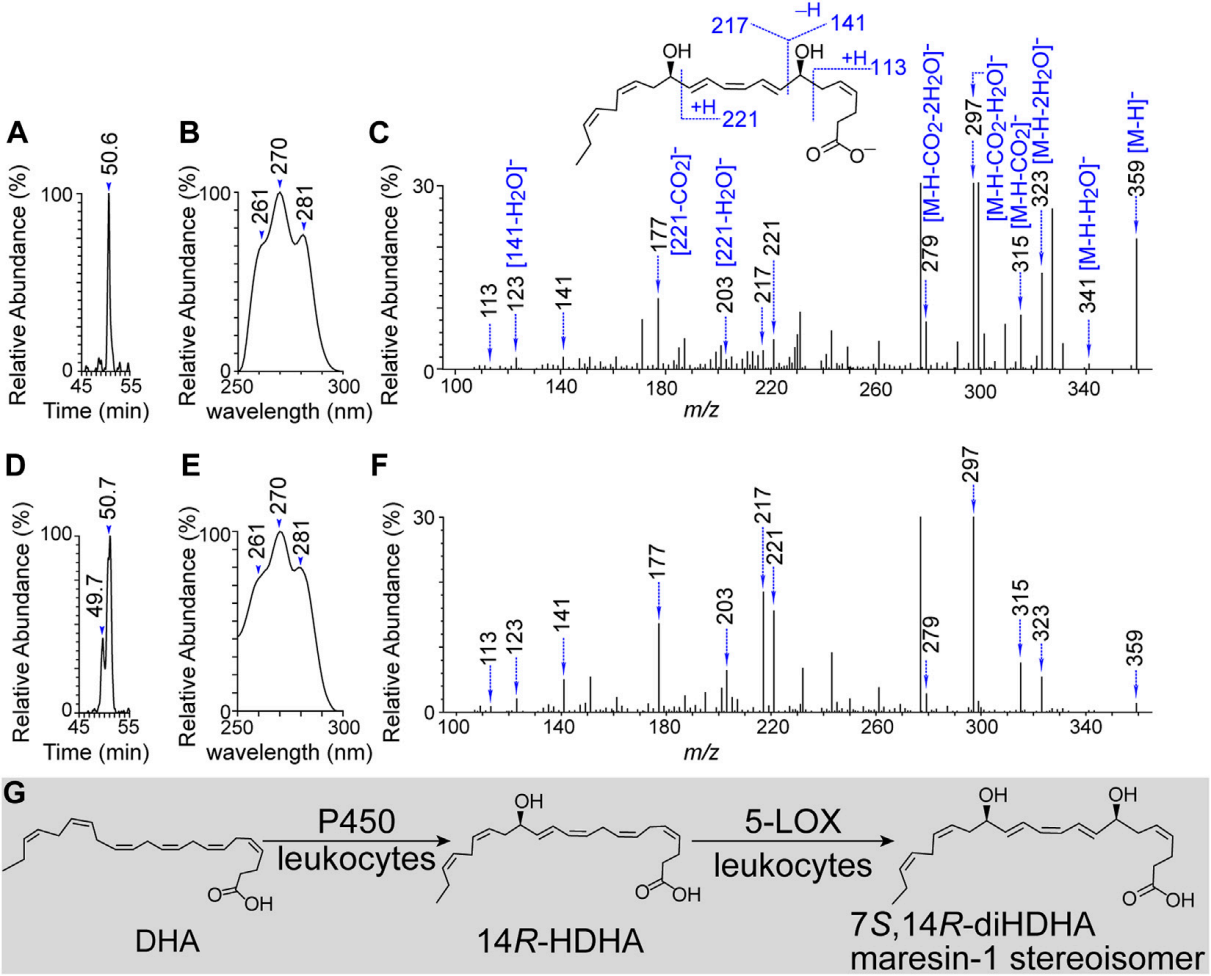
Novel 7*S*,14*R*-dihydroxy-4*Z*,8*E*,10*Z*,*12E*,16*Z*,19*Z*-DHA produced from 14*R*-hydroxy DHA by 5-lipoxygenase or activated human leukocytes. **(A)** Representative aR chiral LC-MS/MS Chromatogram. **(B)** UV spectrum of the flowing chromatographic peak in **(A)**. **(C)** MS/MS spectrum and fragment ion interpretation of the chromatographic peak in **(A)**. **(D)** Representative aR chiral LC-MS/MS Chromatogram. **(E)** UV spectrum of the flowing chromatographic peak in **(D)**. **(F)** MS/MS spectrum and fragment ion interpretation of the chromatographic peak in **(D)**. **(G)** Tentative biogenic pathway for 7*S*,14*R*-diHDHAformation. 7*S*,14*R*-diHDHA was produced by the incubation of 14*R*-hydroxy DHA and 5-lipoxygenase (3 h, 37°C) for **(A–C)**. It was also produced by the incubation of human leukocytes (6 × 10^6^ neutrophils + 5 × 10^5^ monocytes + 3 × 10^6^ lymphocytes) in PBS containing 200 μg 14*R*-HDHA [cultured for 20 min then stimulated by inflammatory factors (10 ng/mL TNFα, 10 ng/mL IL-1β, and 100 ng/mL LPS) for 30 min, 37°C]. The incubation was extracted and analyzed using aR chiral LC-UV-MS/MS as described previously.

14*R*-HDHA forms from DHA by P450 enzymatic conversion, as we observed previously (Hong et al., 2014a). We predicted that 5-LOX or 5-LOX rich leukocytes (el Makhour-Hojeij et al., 1994; Cook-Moreau et al., 2007; Radmark and Samuelsson, 2009; Serhan et al., 2009) convert 14*R*-HDHA to 7*S*,14*R*-dihydroxy-4*Z*,8*E*,10*Z*,*12E*,16*Z*,19*Z*-DHA where the chirality of C-14 is preserved as 14*R*, and the C-7 takes in the hydroxyl at *S*-configuration based on the well-established knowledge of 5-LOX (el Makhour-Hojeij et al., 1994; Cook-Moreau et al., 2007; Radmark and Samuelsson, 2009; Serhan et al., 2009). This prediction was tested positive as we observed 7*S*,14*R-*diHDHA as a single aR-chiral LC peak at 50.6 min (Figure 1A), with its UV spectral triplet possessing a head at 270 nm and two shoulders at 261 and 281 nm representing its conjugated triene (Figure 1B) and its MS/MS spectrum at *m/z* 359 [molecular mass M-H]^-^ finger-printing its 7*S*, 14*R* and double-bond locations (Figure 1C). The MS/MS ions *m/z* 359 [M-H]^-^, 341 [M-H-H_2_O]^-^, 323 [M-H-2H_2_O]^-^, 315 [M-H-CO_2_], 297 [M-H-H_2_O-CO_2_]^-^, and 279 [M-H-2H_2_O-CO_2_]^-^ were consistent with one carboxyl and a molecular weight (M) of 360 Da. The fragment ions *m/z* 217, 141, 123 [141-H_2_O]^-^, and 113 showed a hydroxyl at the 7 position (C_7_). The fragment ions *m/z* 221, 203 [221-H_2_O]^-^, and 177 [221-CO_2_]^-^, demonstrated another hydroxyl at the 14 position (C_14_). These data and our previous observation on P450 formation of 14*R*-HDHA (Hong et al., 2014a) support our prediction that P450 or 5-LOX act together, generating 7*S*,14*R-*diHDHA.

Leukocytes are major blood cells that are essential not only in immunity against infection but also in the protection against or repair of organ degeneration and dysfunction caused by injury, diabetic complication, aging, or other adverse conditions. This is demonstrated by leukocyte production of prohealing resolvins, maresins (Serhan et al., 2009), and/or 14,21-diHDHAs (Lu et al., 2010; Tian et al., 2011b; Hellmann et al., 2012). To further test our first hypothesis, we incubated human leukocytes (monocytes + neutrophils + lymphocytes) in 14*R*-HDHA containing medium as we conducted previously (Tian et al., 2011a), then activated the cells with factors involved in inflammation and organ degeneration. The incubations were then studied using aR chiral LC-UV-MS/MS. 7*S*,14*R-*diHDHA was found to be the peak at the chromatographic retention time (RT) of 50.7 min (Figure 1D) with UV and MS/MS spectra (Figures 1E, F, respectively) matching those of 7*S*,14*R-*diHDHA generated by 5-LOX from 14*R*-HDHA (Figure 1). Of note, a small peak at RT 49.7 min was found to be a 7,14-diHDHA because its UV and MS/MS spectra match those of 7*S*,14*R-*diHDHA in Figures 1E, F. This 7,14-diHDHA is generated from added 14*R*-HDHA as purified leukocytes alone without feeding exogenous substrate did not show detectable 7,14-diHDHA; its 14-hydroxyl should be 14*R*-hydroxyl because the chirality of its 14-hydroxyl is preserved when undergoing such enzymatic conversion. It had RT 49.7 min, shorter than 50.7 min of 7*S*,14*R-*diHDHA, thus, its 7-hydroxyl should be 7*R* because the aR chiral LC RT is shorter for a fatty acid derived *R*-hydroxyl compound than for its *S*-epiomer when other structural features are the same (Figure 1A) on the basis of our extensive studies and publications from others (Schneider et al., 2007; Yin et al., 2007). The 7*R*-hydroxylation is likely to be catalyzed by P450 enzyme(s) in the leukocytes because P450 can catalyze both *R* and *S* hydroxylation. The exact mechanism of this unknown aspect is beyond the scope of this study. In brief, 7*S*,14*R-*diHDHA was the major product, and 7*R*,14*R-*diHDHA was the minor one when feeding leukocytes with 14*R*-DHA. These results and our previous finding that P450 converts DHA to 14*R*-HDHA converged to the biogenic pathway depicted in Figure 1G that human leukocytes produced 7*S*,14*R*-diHDHA, while cell-possessed P450 and 5-LOX catalyzed the biosynthesis.

### 3.2 7*S*,14*R*-diHDHA enhanced MSC function to regulate nonfasting blood glucose levels

**FIGURE 2:**
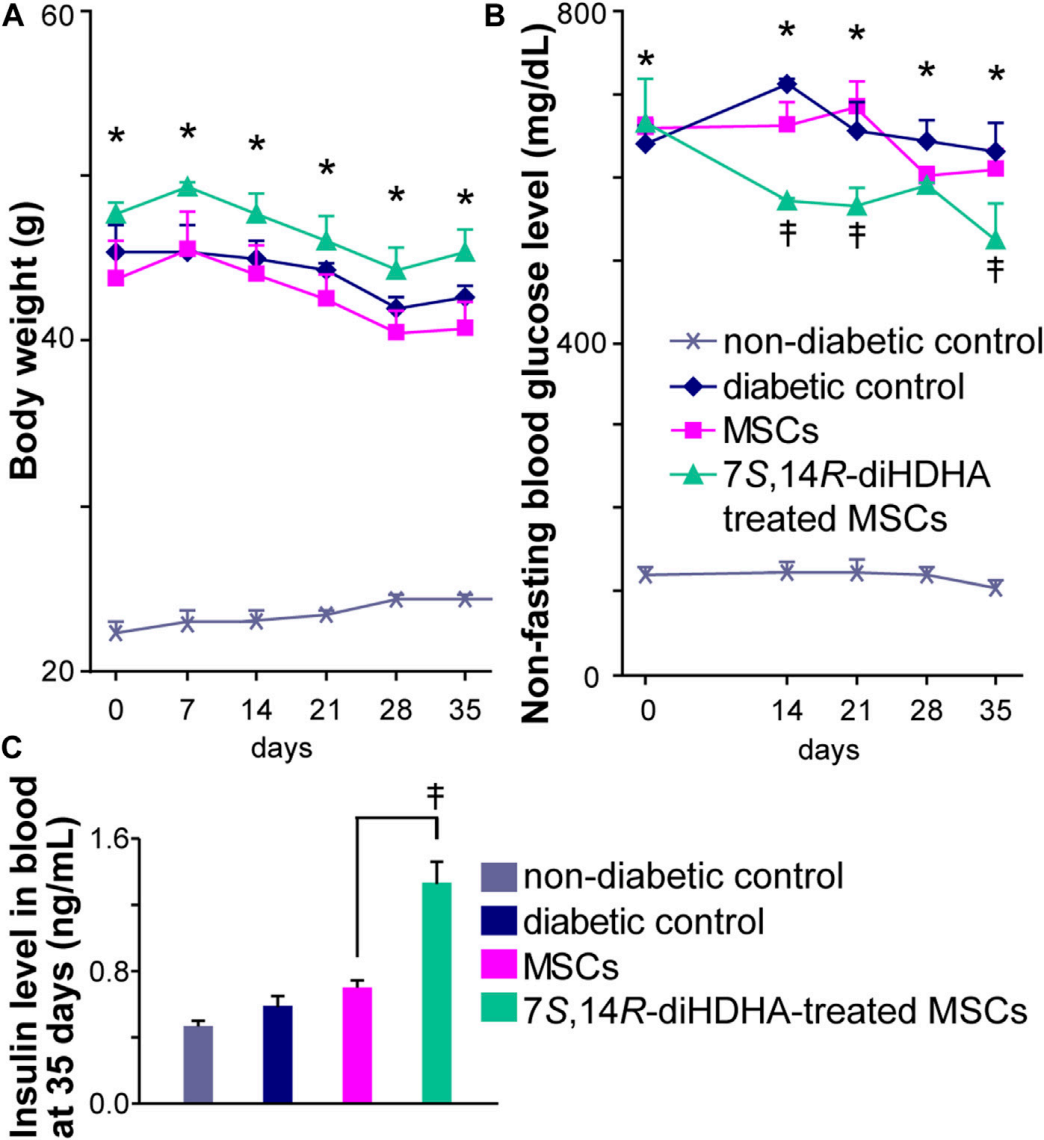
7*S*,14*R*-diHDHA enhanced MSCS function to regulate blood glucose levels. *C57/BL6J* MSCs (1 × 10^6^) with or without treatment of 7*S*,14*R*-diHDHA were infused into *db/db* mice via tail vein every 5 days for 35 days. **(A)** Body weight. **(B)** Nonfasting blood glucose levels. **(C)** Blood insulin levels were measured at the end of treatment (35 days). Data are mean ± SEM, n = 6. *p < 0.05 vs. nondiabetic control, ‡*p* < 0.05 vs. MSCs alone.

The *db/db* mice began to exhibit obesity by 5 weeks of age, and body weight increased rapidly with age (Puff et al., 2011). 16-week old *db/db* mice weighed twice as much as the control *C57BL/6J* mice (45.3 ± 1.7 vs. 22.3 ± 0.7 g, *p* < 0.05, Figure 2A). The weight of non-diabetic control mice increased a little from 16 to 21 weeks old (Figure 2A). In addition, treatment with MSCs or 7*S*,14*R*-diHDHA-treated MSCs had no influence on weight (Figure 2A). The nonfasting blood glucose levels in *db/db* mice were much higher than in normal control *C57BL/6J* mice (640.0 ± 21.4 vs. 120.7 ± 9.3 mg/dL, *p* < 0.05, Figure 2B). At the end of intervention, the blood glucose level did not change in MSC infusion groups, but infusion of 7*S*,14*R*-diHDHA-condictioned MSCs reduced the blood glucose level compared to the untreated MSC group (Figure 2B). Additionally, infusion of 7*S*,14*R*-diHDHA-condictioned MSCs reduced the blood glucose level significantly at day 35 compared to day 0. Blood insulin concentration analysis revealed increased blood insulin levels in the MSC group. Moreover, 7*S*,14*R*-diHDHA augmented the MSC effect on insulin secretion (Figure 2C), thus demonstrating that 7*S*,14*R*-diHDHA improved MSC amelioration of type 2 diabetic mellitus.

### 3.3 7*S*,14*R*-diHDHA treatment promoted the capacity of MSCs to improve glucose tolerance in *db/db* mice

**FIGURE 3:**
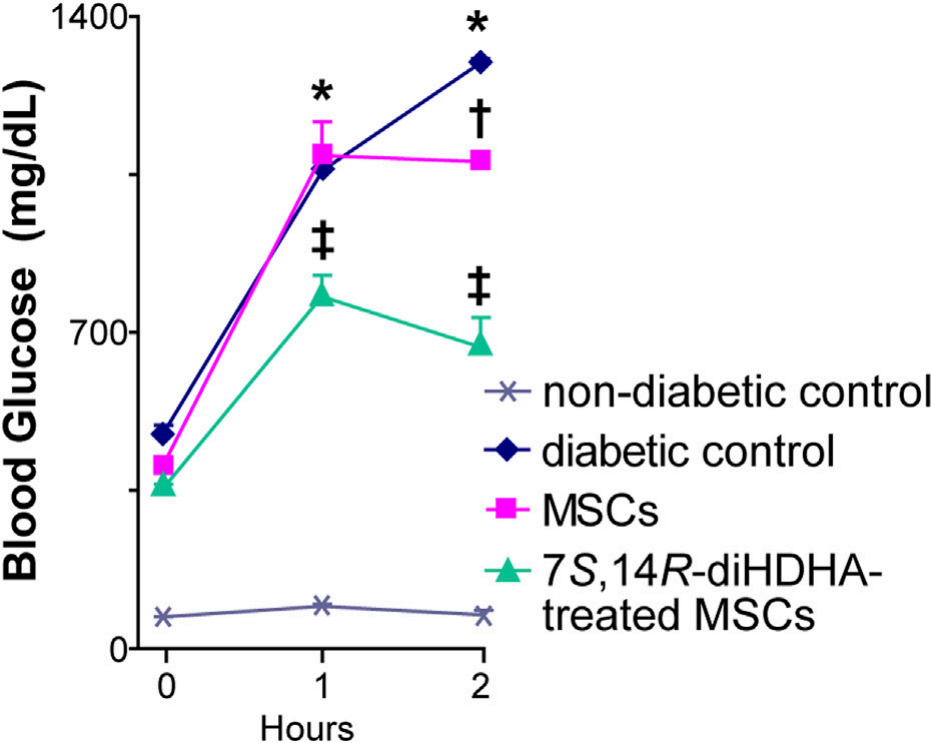
7*S*,14*R*-diHDHA enabled MSCS to promote glucose tolerance in *db/db* mice. *C57/BL6J* MSCs (1 × 10^6^) with or without treatment of 7*S*,14*R*-diHDHA were infused into *db/db* mice via tail vein every 5 days for 35 days. Mice were then injected with glucose (*i.p.,* 2 g/kg body weight) after 8 h of fasting. Blood samples were taken at 0, 1, and 2 h from the tail vein, and blood glucose was measured. Data are mean ± SEM, *n* = 6. *p < 0.05 vs. nondiabetic control; †*p* < 0.05 vs. diabetic control; ‡ *p* < 0.05 vs. MSCs.

IPGTT was performed to determine the effect of the treatments of MSCs and 7*S*,14*R*-diHDHA-treated MSCs on glucose tolerance. The blood glucose concentration at 120 min after glucose load was significantly reduced by MSC treatment (1,078.0 ± 23.4 vs. 1,290.0 ± 12.2 mg/dL, *p* < 0.05, Figure 3). In addition, 7*S*,14*R*-diHDHA-treated MSCs were more effective than untreated MSCs in reducing glucose concentration (667.0 ± 66.8 vs. 1,078.0 ± 23.4 mg/dL, *p* < 0.05, Figure 3). These results suggest that the improvement of glucose tolerance by MSCs and 7*S*,14*R*-diHDHA-treated MSCs was at least in part due to improvement of β-cell function.

### 3.4 7*S*,14*R*-diHDHA induced MSC function to augment the ratio of β-cells and to reduce the ratio of α-cells in pancreatic islets

**FIGURE 4:**
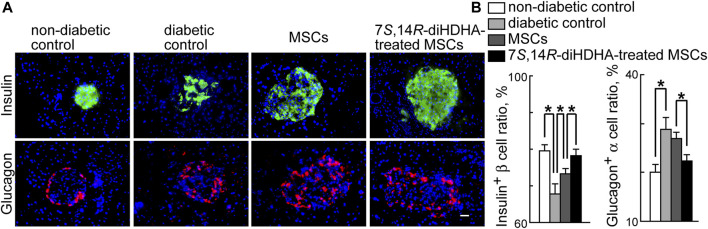
7*S*,14*R*-diHDHA-treated MSCs regulated morphological changes in pancreatic islets. *C57/BL6J* MSCs (1 × 10^6^) with or without treatment of 7*S*,14*R*-diHDHA were infused into *db/db* mice via tail vein every 5 days for 35 days. Pancreas was collected for histological study. **(A)** Representative pancreatic sections showed insulin^+^ β-cells (green), glucagon^+^ α-cells (red) (magnification ×200, scale bar = 50 μm), and nuclei were counterstained with Hochest 33342; **(B)** Quantification of β-cell ratio and α-cell ratio in islets (*n* = 60). The results are mean ± SEM. * *p* < 0.05.

Figure 4 shows typical immunostaining patterns of pancreatic islet tissues in the nondiabetic normal *C57BL/6J* mice, diabetic *db/db* mice, and diabetic *db/db* mice infused with MSCs treated with or without 7*S*,14*R*-diHDHA. The islet of *C57BL/6J* mice, β-cells occupied most parts of the islets and existed at the center, whereas α-cells existed peripherally. However, *db/db* mice generally have hypertrophied pancreatic islets with a mixture of β-cells and α-cells in the islets. In addition, the ratio of β-cells to total islet cells in a pancreatic islet of *db/db* mice is less than that in *C57BL/6J* mice. Although MSC and 7*S*,14*R*-diHDHA-treated MSC infusions cannot change the distribution of β-and α-cells in the islets of *db/db* mice, MSC treatment can increase β-cell ratio compared with diabetic control mice (73.3% ± 1.4% vs. 67.8% ± 2.8%, *p* < 0.05, Figure 4), and 7*S*,14*R*-diHDHA can enhance MSC function to augment this ratio (78.2% ± 1.8% vs. 73.3% ± 1.4%, *p* < 0.05, Figure 4). In addition, 7*S*,14*R*-diHDHA can enhance MSC function to reduce α-cell ratio in islets of *db/db* mice (Figure 4).

### 3.5 7*S*,14*R*-diHDHA enhanced MSC function to decrease the number of macrophages in islets

**FIGURE 5:**
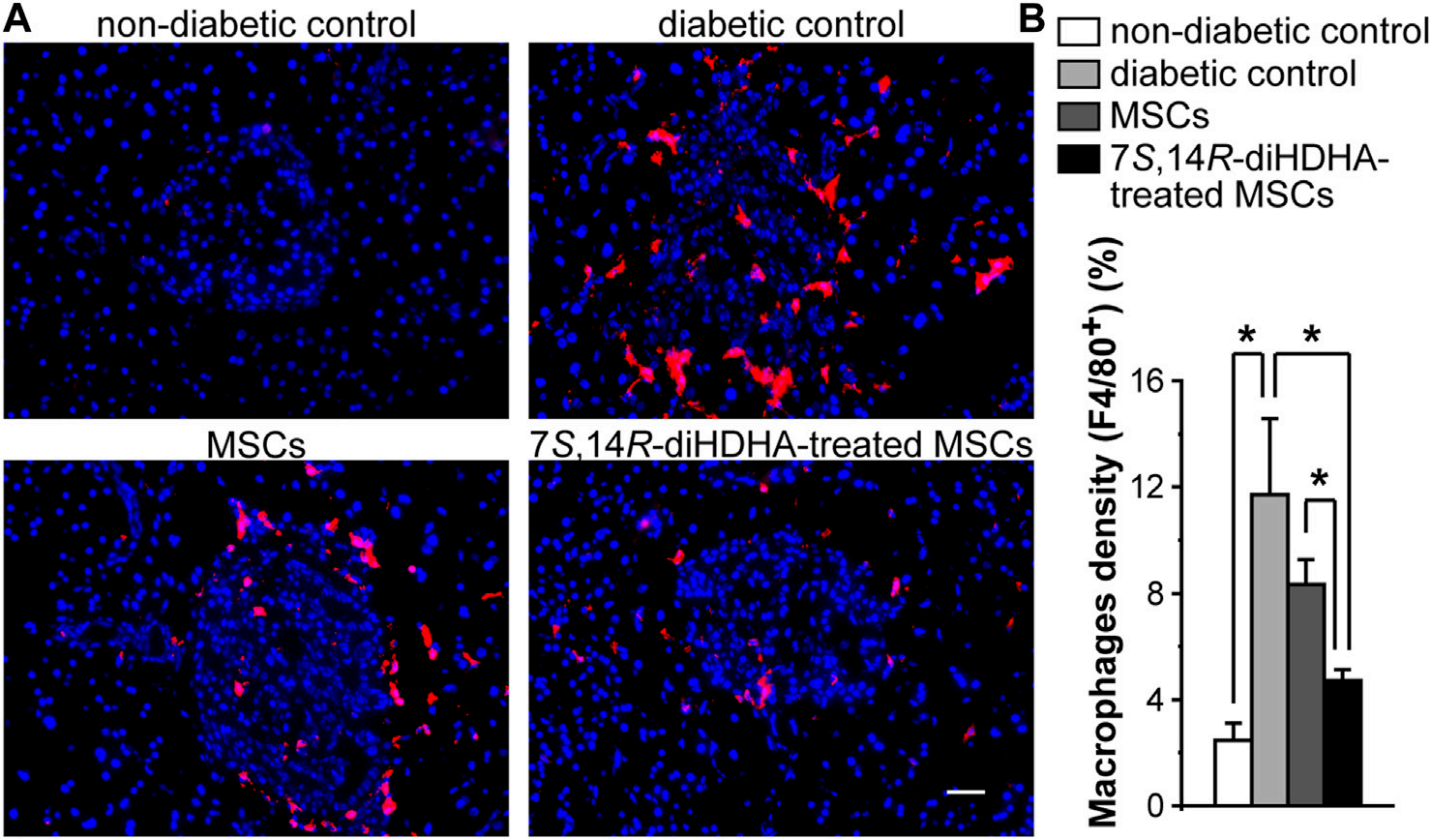
7*S*,14*R*-diHDHA enhanced MSC function to reduce macrophage accumulation in islets. *C57/BL6J* MSCs (1 × 10^6^) with or without treatment of 7*S*,14*R*-diHDHA were infused into *db/db* mice via tail vein every 5 days for 35 days. Pancreas was collected for histological study. **(A)** Typical images of F4/80+ macrophages (red) in pancreatic cryosections (magnification ×200, scale bar = 50 μm), and nuclei were counterstained with Hochest 33342; **(B)** Quantification of macrophage density in islets (*n* = 60). The results are mean ± SEM. *p < 0.05.

Massive macrophage infiltration was observed in the islets of *db/db* mice compared to *C57BL/6J* mice, which suggests that inflammation may contribute to the dysfunctions of β-cells. In MSC-treated *db/db* mice, the number of macrophages in islets was reduced compared with nontreated *db/db* mice (Figure 5). Treatment with 7*S*,14*R*-diHDHA improved the anti-inflammatory action of MSCs.

### 3.6 7*S*,14*R*-diHDHA augmented MSC function to increase min6 β-cell viability and insulin secretion

**FIGURE 6:**
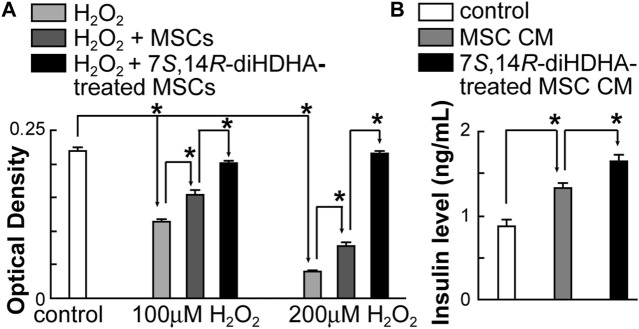
7*S*,14*R*-diHDHA augmented MSC function to increase min6 β-cell viability and insulin Secretion. **(A)** Min6 β-cells were cocultured with MSCs pretreated with or without 7*S*,14*R*-diHDHA and challenged with 100 or 200 μM H_2_O_2_. (*n* = 6). **(B)** Min6 β-cells were treated for 4 h with conditioned medium (CM) of MSCs or 7*S*,14*R*-diHDHA-treated MSCs. Insulin levels were analyzed using ELISA (*n* = 6). The results are mean ± SEM. * *p* < 0.05.

H_2_O_2_ dose-dependently reduced min6 β-cell viability was measured using the MTT method. When cocultured with MSCs, min6 β-cell viability was significantly increased under H_2_O_2_ condition (Figure 6A). Additionally, 7*S*,14*R*-diHDHA augmented the MSC effect on increasing min6 β-cell viability (Figure 6A). Furthermore, when min6 β-cells were treated with MSC conditioned medium, min6 β-cells secreted much more insulin (1.3 ± 0.1 vs. 0.9 ± 0.1 ng/mL, *p* < 0.05; Figure 6B), suggesting that MSCs can generate some trophic factors that trigger insulin release. 7*S*,14*R*-diHDHA-treated MSCs further enhanced insulin secretion from Min6 β-cell (1.7 ± 0.1 vs. 1.3 ± 0.1 ng/mL, *p* < 0.05; Figure 6B), which indicates that 7*S*,14*R*-diHDHA can promote MSCs to generate many more trophic factors acting on min6 β-cells to secrete more insulin.

### 3.7 7*S*,14*R*-diHDHA treatment enhanced MSC secretion of trophic growth factors

**FIGURE 7:**
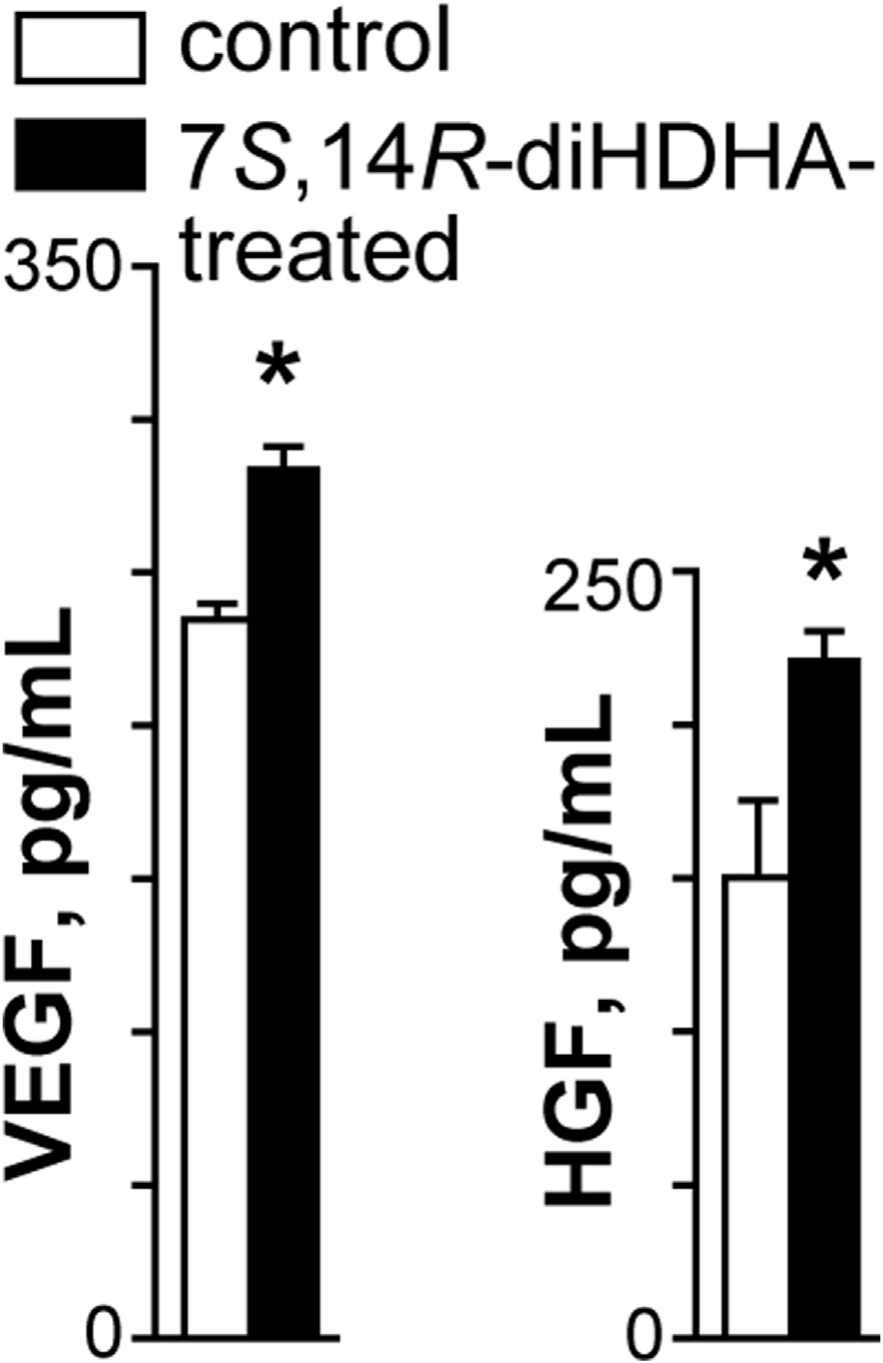
7*S*,14*R*-diHDHA increased the production of VEGF and HGF by MSCs. MSCs (2 × 10^5^) were treated with 7*S*,14*R*-diHDHA (250 nM) for 12 h. The level of VEGF or HGF in the supernatant was analyzed using ELISA kits. VEGF and HGF secretion from MSCs *in vitro* were shown (*n* = 4). The results are mean ± SEM. *p < 0.05 vs. control.

MSCs generate an array of cytokines and growth factors (Wu et al., 2007; Shi et al., 2012). Therefore, we postulated that 7*S*,14*R*-diHDHA treatment enhanced the beneficial effects of MSCs by augmenting their secretion of trophic factors. We observed that 7*S*,14*R*-diHDHA treatment increased the amount of the trophic growth factors VEGF and HGF secreted by MSCs into the cell culture medium (VEGF: 282.5 ± 9.6 vs. 233.0 ± 5.6 pg/mL, *p* < 0.05; 220.7 ± 13.2 vs. 152.3 ± 22.3 pg/mL, *p <* 0.05; Figures 7A, B).

### 3.8 7*S*,14*R*-diHDHA treatment induced MSC function to decrease pericyte loss in the retina

**FIGURE 8:**
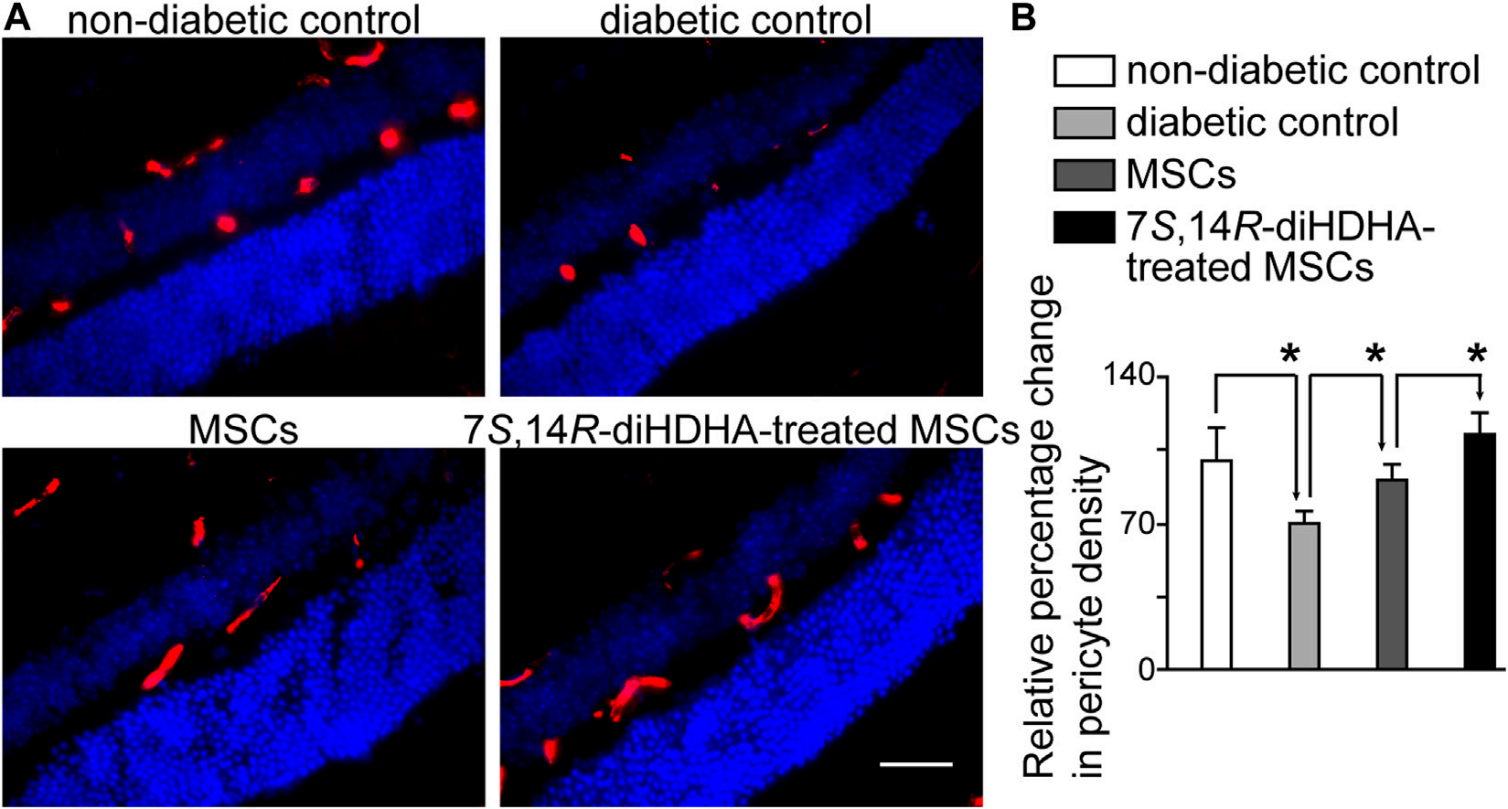
7*S*,14*R*-diHDHA treatment induced MSC function to decrease pericyte loss in retina. *C57/BL6J* MSCs (1 × 10^6^) with or without treatment of 7*S*,14*R*-diHDHA were infused into *db/db* mice via tail vein every 5 days for 35 days. Eyes were collected for histological study**. (A)** Images of α-SMA^+^ pericytes (red) in retinal sections (magnification ×400, scale bar = 50 μm), and nuclei were counterstained with Hochest 33342; **(B)** Quantification of pericyte density in retina (*n* = 30). The results are mean ± SEM. * *p* < 0.05.

Vasoregression is the primary pathogenic response of the retina to chronic hyperglycemia. Loss of capillary pericytes, followed by the formation of acellular, nonperfused capillaries, is an attempted compensation for retinal hypoxia (Hammes et al., 2011). Controlling mellitus can ameliorate diabetic complications; therefore, we want to know whether 7*S*,14*R*-diHDHA-treated MSCs can protect diabetic retinopathy by controlling hyperglycemia. The retinas of *db/db* mice showed a decrease in pericyte density compared with that of the *C57BL/6J* mice (Figure 8). However, MSCs can reduce pericyte loss in *db/db* mice. In addition, 7*S*,14*R*-diHDHA-treated MSCs can further increase the pericyte density compared to untreated MSCs (Figure 8), which suggests that 7*S*,14*R*-diHDHA enhance MSC paracrine function to ameliorate retinal pericyte loss in diabetes through adjusting hyperglycemia and acting pericyte directly.

## 4 Discussion

### 4.1 Leukocytes produced 7*S*,14*R*-diHDHA while P450 and 5-LOX catalyzed the biosynthesis

Taking our current results and previous findings (Hong et al., 2014a) together revealed that leukocytes and their 5-LOX and P450 can produce a novel 7*S*,14*R*-dihydroxy-4*Z*,8*E*,10*Z*,*12E*,16*Z*,19*Z*-DHA or 7*S*,14*R*-diHDHA, a maresin-1 stereoisomer from DHA, as depicted in Figure 1G. We previously found that human P450, including 2D6 (CYP2D6), can catalyze the 14*R*-hydroxylation of DHA, where the contribution of autooxidation is negligible (Hong et al., 2014a). We observed that human leukocytes can convert 14*R*-HDHA to 7*S*,14*R*-diHDHA. Moreover, we observed that human 5-LOX catalyzed the biosynthesis of 7*S*,14*R*-diHDHA from 14*R*-HDHA by generating 7*S*-hydroxy in 14*R*-HDHA. Therefore, 5-LOX and P450 of leukocytes are accountable for the biogenesis of 7*S*,14*R*-diHDHA since 5-LOX is a major LOX and P450 is readily available in leukocytes (el Makhour-Hojeij et al., 1994; Cook-Moreau et al., 2007; Radmark and Samuelsson, 2009; Serhan et al., 2009). The geometries of double-bonds outside of the region from C7 to C14 and the C14 chirality of 14*R*-HDHA are conserved after the transformation to 7,14-diHDHA, based on our results and reported analogous data for eicosanoids (Hong et al., 2003; Capdevila et al., 2005). Thus, 7*S*,14*R*-diHDHA produced by leukocytes or by sequential catalysis of P450 and 5-LOX is 7*S*,14*R*-dihydroxy-4*Z*,8*E*,10*Z*,*12E*,16*Z*,19*Z*-DHA.

### 4.2 Harnessing mesenchymal stem cells by 7*S*,14*R*-diHDHA to ameliorate diabetic mellitus and retinal pericyte loss

The etiologies for type 1 and type 2 diabetes differ. Type 1 diabetes is a chronic autoimmune disease hallmarked by immune-mediated destruction of the pancreatic β-cells. T lymphocytes are aberrantly activated by the antigen-presenting cells in the pancreatic draining lymph nodes, and the activated T lymphocytes then circulate, target, invade the islets and destroyed the β-cells (Mathis et al., 2001; Pugliese, 2017). However, type 2 diabetes, hallmarked by underlying insulin resistance, is also characterized by defects in glucose-responsive insulin secretion in addition to an eventual decline in β-cell function (Rhodes, 2005; Dludla et al., 2023), and oxidative stress caused by hyperglycemia is considered to be the main reason leading β-cell apoptosis (Cerf, 2013; Inaishi and Saisho, 2020; Miranda et al., 2020; Eguchi et al., 2021; Dinic et al., 2022; Dludla et al., 2023).

MSCs have multiple differentiation and paracrine functions (Miklosz and Chabowski, 2023). It has been confirmed that MSC transplantation into STZ-induced type 1 diabetic mouse and NOD models can enhance islet regeneration, lower blood sugar, and increase blood insulin levels (Jurewicz et al., 2010; Gabr et al., 2013; Yousef et al., 2022; Carlsson et al., 2023). Although MSCs can be recruited to the pancreas, less than 3% of cells migrate to the pancreas (Lee et al., 2006; Preda et al., 2021) confirmed the direct differentiation capacity of MSCs is not the main mechanism, which suggests that the paracrine function may confer tissue-repairing function to MSCs. MSCs can generate many bioactive factors, such as TGF-α, HGF, IDO, PGE_2_, which play important roles in modulating immuno-cell functions, such as inhibiting T cell proliferation, dendritic cell activation, and inflammatory cytokine generation (Soleymaninejadian et al., 2012; Jiang and Xu, 2020). On the basis of generation immunomodulating factors, MSCs inhibit the onset of type 1 diabetes and reverse β-cell functions (Vija et al., 2009; Jayasinghe et al., 2022). However, type 2 diabetes is different from type 1 diabetes, and immunomodulating functions may not be the reason for MSCs repairing damaged β-cells in type 2 diabetes. Increased leukocyte infiltration is associated with the onset of type 2 diabetes (Ford, 2002). Depletion of macrophages by clodronate in liposomes reduced pancreatic invasion of macrophages and destruction of islet β-cells (Inokuchi et al., 2009; Chan et al., 2019). Our results revealed that MSCs can inhibit macrophage infiltration into islets. The increased oxidative stress observed in islets of diabetic states seems to be one of the main factors of deteriorated β-cell function. We have found that MSCs can inhibit macrophage generation of the inflammatory cytokines TNF-α and ROS (Tian et al., 2012), which could implicate the potential anti-inflammatory functions of MSC protecting β-cell functions in type 2 diabetes. This implication could be confirmed by the levels of inflammation markers, such as TNFα, IF1, and IF6, which is an important direction that we need to pursue in the future.

The islet capillary network is about five times denser than the capillary network of exocrine tissue. The enriched vascularity meets the requirement for the quick secretion of insulin from β-cell in response to blood glucose levels (Konstantinova and Lammert, 2004). In diabetic *db/db* mice, the number of endothelial cells was reduced compared with normal nondiabetic mice. VEGF is one of the most important angiogenic cytokines (Li et al., 2003). We have confirmed that MSCs can generate VEGF and promote angiogenesis in wounds, which promotes wound healing in *db/db* mice (Tian et al., 2011a). MSCs promote vascularity in islets of *db/db* mice may also through generating VEGF.

DHA is a typical ω-3 long-chain poly-unsaturated fatty acid, it can be transformed enzymatically into potent inflammation-resolving lipid mediators, such as Resolvin D series (Spite and Serhan, 2010), neuroprotectin/protectins (Hong et al., 2003; Mukherjee et al., 2004; Zhao et al., 2011; Hong et al., 2014b), maresins (Serhan et al., 2009; Martinez-Fernandez et al., 2021), maresin-likes (Hong et al., 2014a), and 7*S*,14*R*-diHDHA. We also found a DHA-derived lipid mediators 14*S*,21*R*-diHDHA, which can enhance MSC functions to promote wound healing and repair acute kidney injury (Tian et al., 2011a; Tian et al., 2012). 7*S*,14*R*-diHDHA is a new lipid mediator that has a potential anti-inflammatory function. In this study, we observed that 7*S*,14*R*-diHDHA can promote MSC paracrine function to generate VEGF and HGF. HGF has anti-inflammatory properties; it can increase IL-10 levels and reduce IL-1β levels in pancreatitis or diabetes (Warzecha et al., 2004; Oliveira et al., 2018; Greer et al., 2022). These data suggest that 7*S*,14*R*-diHDHA promotes MSC functions to ameliorate diabetic mellitus mainly by enhancing MSC paracrine functions. These results are consistent with reports that maresins promote the beneficial bioactions of stem cells in the treatment of diseases (Martinez-Fernandez et al., 2021). Maresin 1 regulates insulin signaling in human adipocytes as well as in adipose tissue and muscle of lean and obese mice (Martinez-Fernandez et al., 2021). Maresin 1 inhibits hyperglycemia-induced ferroptosis (Li et al., 2023).

Diabetic retinopathy is a clinically well-defined, sight-threatening, chronic microvascular complication that eventually affects virtually all patients with diabetes. Diabetic retinopathy is characterized by gradually progressive alterations in the retinal microvasculature, which start with the loss of the two cellular components of retinal capillaries: the pericyte, a vessel support cell, and the endothelial cell, and pericytes disappear before endothelial cells start to vanish (Hammes et al., 2004; Eshaq et al., 2017; Gange et al., 2021). *db/db* mice develop hyperglycemia starting at 8-week age as a result of excessive food consumption (Kodama et al., 1994; Dalboge et al., 2013). They show early signs of diabetic retinopathy, such as thickening of capillary basement membrane at 22 weeks (Clements et al., 1998) and loss of pericytes in retinas at 26 weeks, followed by endothelial cell loss at 34 weeks (Midena et al., 1989). In our study, pericyte loss occurred as early as 22 weeks of age in *db/db* mice. MSCs can inhibit pericyte loss. Moreover, 7*S*,14*R*-diHDHA-treated MSCs further markedly increased pericyte density compared with untreated MSCs. This suggests that 7*S*,14*R*-diHDHA enhances MSC paracrine function to ameliorate retinal pericyte loss in diabetes by adjusting for hyperglycemia and/or acting pericyte directly. This enhancement is likely to occur through MSC secretion of VEGF, as 7*S*,14*R*-diHDHA can significantly promote MSC production of VEGF (Figure 7), and VEGF is thought to be an important cytokine inducing vasoregeneration in diabetic retinopathy (Hammes et al., 2011). When MSCs were infused into diabetic mice, MSCs have an anti-inflammatory function, reducing oxidative stress. Under this condition, VEGF or HGF generated from MSCs may trigger the PI3K/AKT signaling pathway and maintain retinal vascular integrity (Abid et al., 2004; Yun, 2021), while 7*S*,14*R*-diHDHA treatment can enhance MSC function via this pathway. The mechanism for the amelioration of diabetic damage of retinal pericytes by 7*S*,14*R*-diHDHA-treated MSCs is also likely attributable to their amelioration of diabetes, especially diabetic hyperglycemia.

### 4.3 Future directions

The scope of this pilot study provides multiple opportunities for future research. 5-LOX could convert DHA to 7*S*-hydroxyl DHA first, followed by P450 14*R*-hydroxylation in sequential enzymatic catalysis to generate 7*S*,14*R*-diHDHA from DHA. However, the exact P450 enzymes involved in 14*R*-hydroxylation are unknown. The appearance of 7*S*,14*R*-diHDHA in the blood, pancreas, and other organs *in vivo* in animals and humans under healthy and diabetic conditions is likely to be transient, which needs to be studied systematically at different time points. We will study the actions of 7*S*,14*R*-diHDHA alone. Future studies should also evaluate the effect of our treatments on the other aspects of diabetes and on diabetic retinopathy, as well as conduct assessments, including cell cycle analysis, characterization of biomarkers, interactions with immune cells, and morphological evaluations of mouse and human MSCs with and without 7*S*,14*R*-diHDHA treatment. Our future study will be performed *in vitro* to determine if the MSCs with and without the treatment by this novel lipid mediator can affect immune cells, including lymphocyte inhibition assay, migration and cytokine production of lymphocytes, monocytes, neutrophils, eosinophils, and basophils. We will compare how mouse MSCs from fat tissue and bone marrow react to this novel lipid mediator to delineate systematically if one source is better than the other or similar as this is another important milestone to translate our work to clinical application. We observed that only when MSCs were treated with 7*S*,14*R*-diHDHA could increase insulin level and reduce blood sugar level. This may be due to the low dose of MSCs that we used. In the future, we will apply high doses of MSCs to treat diabetes. In addition, type 2 diabetes is insulin resistance. The level of blood sugar is not only related to the level of insulin, but also related to the regulatory functions of the liver and other organs. 7*S*,14*R*-diHDHA-treated MSCs may not only promote the secretion of insulin, but also have the functions of improving insulin resistance, improving the regulatory functions of the liver and other organs, and reducing the level of blood sugar in diabetes. We will further detect the function of the liver and other organs in regulating blood sugar.

## 5 Conclusion

We identified a novel DHA-derived 7*S*,14*R*-dihydroxy-4*Z*,8*E*,10*Z*,*12E*,16*Z*,19*Z*-DHA (7*S*,14*R*-diHDHA), a maresin-1 stereoisomer, biosynthesized by leukocytes and related P450 and 5-LOX enzymes (el Makhour-Hojeij et al., 1994; Cook-Moreau et al., 2007; Radmark and Samuelsson, 2009). 7*S*,14*R*-diHDHA promoted MSC paracrine functions in *db/db* mice *in vivo* to ameliorate diabetic mellitus through improving β-cell function, lowering blood sugar, improving glucose tolerance, and increasing blood insulin levels. It augmented the proportion of β-cells and reduced that of α-cells to total islet pancreatic cells, and decreased the number of macrophages in islets in *db/db* mice *in vivo*. Also, the lipid mediator enhanced MSC function to increase min6 β-cell viability and insulin and MSC secretion of trophic growth factors. Furthermore, 7*S*,14*R*-diHDHA treatment induced MSC function to decrease pericyte loss in the retinas of diabetic *db/db* mice *in vivo*. Taken together, pretreatment of MSCs with 14*S*,21*R*-diHDHA or related LMs or structural mimics may offer a new clinical strategy for improved treatment of diabetic mellitus and diabetic complications.

## Data Availability

The original contributions presented in the study are included in the article/supplementary material, further inquiries can be directed to the corresponding authors.
